# Long‐Term Outcomes of Class III Malocclusion Interception With Maxillary Expansion and Protraction: A 9‐Year Follow‐Up Case Report

**DOI:** 10.1155/crid/2771248

**Published:** 2026-05-26

**Authors:** Jenny Angélica Saldarriaga-Valencia, Ary dos Santos-Pinto, Carlos M. Ardila

**Affiliations:** ^1^ Pediatric Dentistry Department, Faculty of Dentistry, CES University, Medellín, Antioquia, Colombia, ces.edu.co; ^2^ Department of Basic Sciences, Faculty of Dentistry, Universidad de Antioquia U de A, Medellín, Antioquia, Colombia, udea.edu.co; ^3^ Department of Orthodontics and Pediatric Dentistry, Araraquara School of Dentistry, Universidade Estadual Paulista-UNESP, Araraquara, Brazil; ^4^ Department of Periodontics, Saveetha Dental College, Saveetha Institute of Medical and Technology Sciences, SIMATS, Saveetha University, Chennai, Tamil Nadu, India, saveetha.com; ^5^ Department of Basic Sciences, Biomedical Stomatology Research Group, Faculty of Dentistry, Universidad de Antioquia U de A, Medellín, Antioquia, Colombia, udea.edu.co

**Keywords:** Class III malocclusion, fixed orthodontics, long-term follow-up, maxillary deficiency, maxillary protraction, skeletal dysplasia interception

## Abstract

This case report presents the successful long‐term management of a Class III malocclusion in an 8‐year‐old male patient treated with maxillary protraction facemask therapy (MPFM) combined with Hyrax‐type maxillary expansion (HME), followed by a 9‐year follow‐up period. The patient presented with a Class III malocclusion (Angle classification), anterior and posterior crossbite, maxillary hypoplasia, and compromised facial esthetics. The treatment protocol consisted of 12 months of MPFM with HME, followed by retention using a modified Type III activator and chin cup, and subsequent fixed orthodontic therapy for final occlusal refinement. Cephalometric evaluation demonstrated significant skeletal improvements, with the ANB angle increasing from −1.0° to 3.1°, the SNA angle increasing from 82.0° to 84.2°, and the SNB angle decreasing from 83.0° to 81.1°. A marked increase in maxillary incisor inclination was observed, with the U1–NA angle increasing from 24.5° to 33.8°. Mandibular incisors exhibited minor linear positional changes (L1–NB, millimeters) and a reduction in angular inclination (L1.NB), indicating dentoalveolar adaptation during treatment. The overjet correction was particularly notable, improving from −1.7 to 3.4 mm, whereas enhanced lip support contributed to improved facial esthetics. The 9‐year follow‐up examination revealed excellent stability in maxillomandibular relationships, with maintained molar and incisor positions as well as stable overjet and overbite measurements. This case demonstrates that early intervention combining HME and MPFM can effectively correct Class III malocclusion with maxillary hypoplasia and mandibular prognathism. However, the importance of continued growth monitoring following active treatment cannot be overstated, as residual growth patterns may influence long‐term stability. These findings support the use of interceptive orthopedic approaches in growing Class III patients while emphasizing the need for extended follow‐up through the remaining growth period. This stability is interpreted considering growth‐related drift: the postpubertal reduction in ANB and overjet reflects residual mandibular growth rather than relapse, whereas stable incisor proclination contributed to occlusal maintenance.

## 1. Introduction

Pediatric dentists and orthodontists frequently encounter patients in mixed dentition presenting developing skeletal Class III malocclusion, a challenging condition requiring careful diagnosis of craniofacial growth patterns, occlusal characteristics, and associated functional problems [1, 2]. The optimal timing for intervention depends on several factors, particularly the degree of skeletal and dental discrepancy and the severity of occlusal and masticatory dysfunction [1, 3].

Class III malocclusion represents a sagittal discrepancy resulting from maxillary hypoplasia, mandibular prognathism, or a combination of both. Patients with maxillary hypoplasia typically exhibit reduced transverse and anteroposterior dimensions of the upper arch, often accompanied by lingually inclined maxillary incisors and labially inclined mandibular incisors due to restricted growth of the nasomaxillary complex [1–3]. The etiology of this condition is multifactorial, involving both hereditary and environmental influences. Strong genetic associations have been documented, particularly with certain X chromosome aneuploidies [4–6], whereas environmental contributors include enlarged tonsils, oral breathing habits, congenital anomalies, hormonal imbalances, mandibular protrusion habits, postural factors, trauma, systemic diseases, and irregularities in permanent tooth eruption or premature loss of primary teeth [2].

Epidemiological studies reveal varying prevalence rates across ethnic groups: approximately 3%–5% in Caucasian populations [3], increasing to 14% among Asians [4]. In American populations, the incidence averages 5%, whereas Latin American adolescents show a higher prevalence of 9.1% [5]. Thilander et al. reported a 3.7% occurrence rate in their study of 4724 Latin American children and adolescents [6]. The phenotypic presentation varies, with some studies identifying maxillary hypoplasia as the predominant feature (occurring in 25% of cases) [3], whereas others report combinations of maxillary deficiency with normal or mildly prognathic mandibles. Guyer et al. found that 25% of their sample exhibited pure maxillary retrusion with mandibular prognathism [1]. Cephalometrically, maxillary hypoplasia manifests through reduced ANB and SNA angles, decreased maxillary length, and diminished A–Nperp measurements. These skeletal deficiencies often lead to characteristic dental compensations, including lingually inclined maxillary teeth and labially tipped mandibular anterior teeth in anterior crossbite relationships [1–3].

The functional consequences of Class III malocclusion include abnormal masticatory patterns with anterior mandibular displacement due to dental interferences. Early interceptive treatment can prevent damage to oral tissues and significantly mitigate severe maxillomandibular disharmonies [7]. Current evidence suggests that orthopedic forces applied during active craniofacial growth, particularly in early mixed dentition, can effectively improve Class III malocclusions. The most documented successful approach combines maxillary protraction with maxillary expansion [8].

The biomechanical rationale for maxillary protraction involves applying controlled forces to the circummaxillary sutures, which remain minimally fused during growth periods, thereby stimulating bone deposition at these sites [4–6]. Contemporary protocols typically combine protraction facemask therapy with rapid maxillary expansion using devices such as the Hyrax appliance. This technique has evolved significantly since its first description over a century ago, with Delaire′s 1976 facemask design and Petit′s 1983 modifications (which increased applied forces while reducing treatment duration) representing major advancements [3].

Cephalometric studies demonstrate multiple effects of facemask therapy: anterior maxillary displacement with forward movement of maxillary teeth, molar extrusion and mesialization, mandibular downward‐backward rotation, proclination of upper incisors, retroclination of lower incisors, and significant angular changes including increased SNA and ANB angles with decreased SNB measurements. Soft tissue changes include anterior displacement of point A, posterior movement of point B, increased facial convexity (NaPg angle), vertical dimension augmentation, and anterior palatal constriction [6–8]. Maxillary expansion enhances these effects by mobilizing the intermaxillary and circummaxillary sutures, creating favorable conditions for protraction forces transmitted through the Hyrax appliance to the maxillary molars, with counterforces distributed to the forehead and chin [4–6].

This article presents a case report documenting successful long‐term management of mixed dentition Class III malocclusion using combined maxillary protraction facemask therapy (MPFM) and Hyrax‐type maxillary expansion (HME), followed by comprehensive orthodontic treatment and 9 years of follow‐up observation.

## 2. Case Report

An 8‐year‐old male patient presented to the Pediatric Dental Clinics at CES University in Medellín, Colombia, accompanied by his parents, with the chief complaint of an evident anterior crossbite. Clinical examination revealed an Angle Class III malocclusion characterized by anterior and bilateral posterior crossbites, associated with maxillary hypoplasia and compromised facial esthetics. The etiology of the malocclusion was primarily attributed to genetic factors, consistent with the reported 3.7% prevalence of Class III malocclusion in the Colombian population [6]. No significant environmental contributing factors were identified through clinical and radiographic evaluation; however, a positive family history of Class III malocclusion was confirmed during anamnesis.

Facial analysis demonstrated a predominantly horizontal growth tendency, characterized by maxillary deficiency combined with relative mandibular excess. The patient presented a mildly concave facial profile, with a normal nasolabial angle and a shallow mentolabial fold. Despite the skeletal Class III relationship, the severity of the facial profile was attenuated, likely due to dentoalveolar compensation and soft tissue characteristics rather than a marked maxillomandibular discrepancy.

Cephalometric evaluation using the Steiner analysis was performed at all stages of follow‐up, using normative reference values (Sn–GoGn mean = 32.6°, SD = 5.2°). Although the initial SN–GoGn value was within the normative range, longitudinal assessment revealed a progressive reduction of this angle, becoming particularly evident at the postpubertal stage. This evolution supports the classification of the patient as having a hypodivergent growth pattern with a predominantly horizontal mandibular growth tendency.

**Figure 1:**
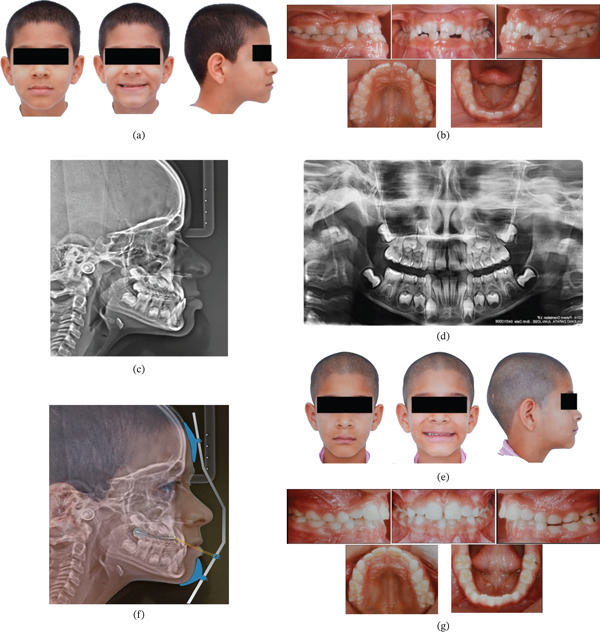
(a) Pretreatment facial photographs showing characteristic features of Class III malocclusion including mildly concave profile and deficient maxillary projection. (b) Pretreatment intraoral photographs demonstrating anterior and posterior crossbites, and Class III molar relationship. (c) Pretreatment lateral cephalometric radiograph revealing skeletal Class III pattern with maxillary deficiency and mandibular prognathism. (d) Pretreatment panoramic radiograph showing mixed dentition with all permanent teeth developing normally. (e) Clinical application of Petit‐type facemask with protraction elastics. (f) Facial photographs following maxillary expansion and protraction therapy showing improved facial balance. (g) Occlusal photographs after active orthopedic treatment demonstrating correction of crossbites.

From a diagnostic standpoint, this growth pattern is clinically relevant because the ANB angle is influenced by craniofacial morphology, including the anteroposterior position of Nasion and vertical jaw rotation. In hypodivergent patients, mandibular rotation may reduce the ANB value and accentuate the apparent Class III relationship. For this reason, complementary assessments such as the Wits appraisal are useful to provide a more reliable evaluation of sagittal discrepancies. Clinical signs of maxillary deficiency included underdeveloped zygomatic projection and reduced orbital support, whereas mandibular prognathism was reflected by increased lower lip length and chin prominence. Vertical facial proportions were consistent with a mild brachyfacial pattern, without evident facial asymmetry. Smile esthetics were compromised by reduced maxillary incisor display and excessive chin prominence (Figure 1).

Occlusal evaluation confirmed maxillary hypoplasia with anterior and bilateral posterior crossbites. The dental arches were narrow but proportionate, with erupted permanent first molars and maxillary central incisors, and the right lateral incisor in the eruptive phase. A mesial step relationship was observed between the second primary molars, and the maxillary canines were positioned in a Class I relationship. The patient exhibited a negative overjet with a clinically normal overbite (Figure 1)b.

Based on the combined clinical and cephalometric findings, the malocclusion was diagnosed as a true skeletal Class III rather than a functional (pseudo‐) Class III. No clinically significant centric relation–centric occlusion discrepancy was identified. Reduced maxillary length (Co–A) and mandibular prominence relative to the cranial base support a structural skeletal etiology, further reinforced by a positive family history and the absence of functional or environmental contributing factors.

Cephalometric analysis revealed a sagittal skeletal discrepancy between the maxilla and mandible, expressed clinically as anterior crossbite and negative overjet, and radiographically as maxillary retrusion combined with mandibular prominence (Figure 1)c. Although the ANB angle was −1.0°, suggesting a mild sagittal discrepancy, the Wits appraisal of −7.9 mm indicated a more pronounced anteroposterior skeletal disharmony. This discrepancy can be explained by the sensitivity of the ANB angle to the anteroposterior position of Nasion and to vertical jaw rotation, particularly in patients with a horizontal growth tendency. In contrast, the Wits appraisal relates the maxilla and mandible directly to the occlusal plane and often provides a more accurate representation of sagittal skeletal relationships in such growth patterns.

The pretreatment panoramic radiograph showed a mixed dentition stage consistent with the patient′s chronological age, with normal development and positioning of the permanent tooth germs. No radiographic signs of agenesis, supernumerary teeth, root resorption, or other pathological alterations were observed. Overall dental development and eruption sequence appeared within normal limits, providing a favorable baseline for orthopedic intervention (Figure 1)d.

Detailed cephalometric measurements demonstrated a maxilla positioned within normal limits relative to the cranial base (SNA and A–Nperp), but with clear evidence of maxillary underdevelopment, as reflected by reduced Ans–Pns and Co–A lengths. The mandible was protruded relative to the cranial base (increased SNB and Pog–Nperp), with normal mandibular body length but increased effective mandibular length (Co–Gn). The resulting skeletal Class III pattern was further supported by negative ANB and Wits values. No clinically significant vertical incisor discrepancy was observed (Table 1).

**Table 1:** Cephalometric values during each phase of treatment.

Cephalometric values					
Measurement	Initial	Posttreatment	Evolution	Long term	Postpubertal
SNA	82.0°	84.2°	85.0°	84.3°	86.4°
SNB	83.0°	81.1°	83.3°	83.0°	85.5°
ANB	−1.0°	3.1°	1.7°	1.3°	0.9°
Witts	−7.9 mm	−0.8 mm	−0.8 mm	−1.0 mm	1.2 mm
A–Nperp	2.4 mm	3.3 mm	3.4 mm	3.8 mm	1.1 mm
Pog–Nperp	6.3 mm	3.4 mm	4.9 mm	3.2 mm	3.0 mm
Co–A	72.6 mm	75.2 mm	76.5 mm	77.2 mm	88.4 mm
Co–Gn	96.4 mm	97.3 mm	98.5 mm	99.5 mm	115.6 mm
SNGoGn	32.0 °	30.0 °	31.5 °	27.8 °	23.8 °
Ans–Me	49.8 mm	50.3 mm	49.3 mm	47.9 mm	57.1 mm
U1–NA	2.6 mm	4.2 mm	3.8 mm	3.9 mm	4.9 mm
U1.NA	24.5 °	33.8 °	32.0 °	29.7 °	29.6 °
L1–NB	2.4 mm	2.0 mm	2.6 mm	2.0 mm	3.0 mm
L1.NB	24.4 °	12.4 °	21.0 °	19.5 °	21.4 °
Overjet	−1.7 mm	3.4 mm	6.7 mm	4.2 mm	2.5 mm
Overbite	1.9 mm	2.8 mm	1.3 mm	3.6 mm	3.4 mm

## 3. Treatment Objectives and Alternatives

The comprehensive diagnostic evaluation established a diagnosis of skeletal Class III malocclusion combining mild maxillary deficiency with mandibular excess, accompanied by anterior and posterior crossbites. The treatment plan focused on crossbite correction and skeletal relationship management through MPFM combined with HME. Following detailed discussion of treatment alternatives, risks, and benefits with the patient and parents, written informed consent was obtained. This consent specifically included permission for the publication of the patient′s identifiable images within this manuscript.

While chin cup therapy represents a viable alternative for managing maxillomandibular discrepancies—aiming to restrain mandibular growth, influence ramus vertical development, and affect gonial angle closure—its effectiveness in producing permanent mandibular dimensional changes remains limited. This limitation stems from the tendency for maxillary growth to compensate following anterior crossbite correction. The rationale for selecting maxillary protraction instead emphasized the mechanical restriction imposed by anterior crossbite on maxillary forward growth, with early intervention promoting more favorable growth patterns.

### 3.1. Treatment Progress

Details regarding appliance sequence, duration, use of fixed orthodontic appliances, and retention protocol were added.

The treatment protocol followed Turley′s established approach [9], incorporating rapid palatal expansion using a Hyrax appliance banded to first permanent molars, supplemented with labial hooks at canine positions for facemask attachment. The expansion screw was activated 0.25 mm daily for 20 days, continuing until achieving proper anteroposterior correction. A Petit‐type facemask delivered 300–500 g (16 oz) of force per side at 30°–40° to the occlusal plane, worn 14–16 h daily (Figure 1)e.

Twelve‐month evaluation demonstrated successful correction of the skeletal Class III relationship, with intentional overcorrection of anterior crossbite to minimize relapse potential. Significant improvements were noted in maxillary incisor position, interincisal relationship, upper lip projection, and overall facial esthetics. The retention phase employed a modified Type III activator combined with chin cup therapy to control residual mandibular growth, with parents counseled regarding the necessity for 4‐year monitoring during continued growth (Figure 1f,g). Following completion of the orthopedic phase and growth monitoring, comprehensive fixed orthodontic appliances were placed to achieve final alignment and occlusal refinement.

**Figure 2:**
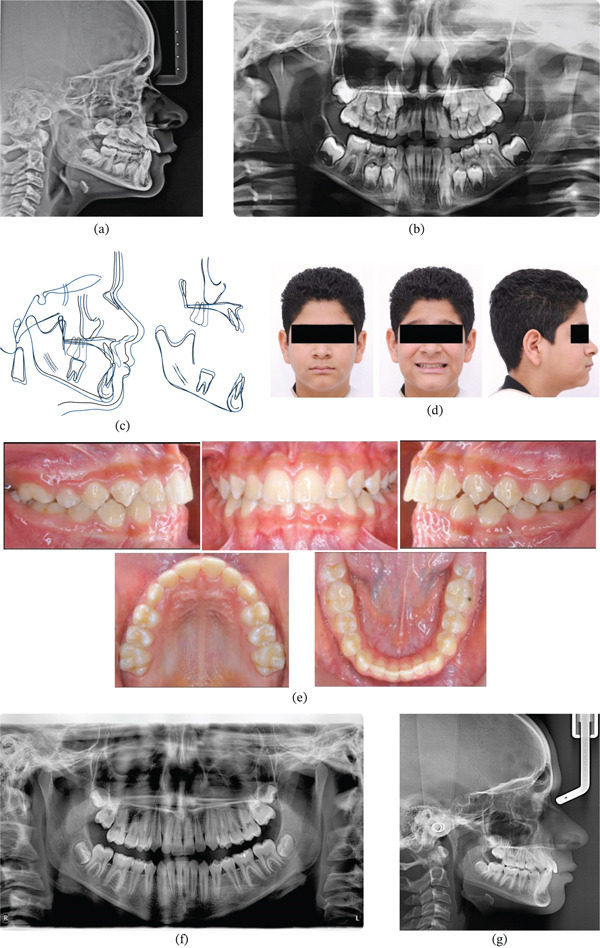
(a) Postprotraction lateral cephalogram showing improved maxillomandibular relationship. (b) Postprotraction panoramic radiograph revealing dental development progression. (c) Cephalometric superimposition comparing pre‐ and postprotraction skeletal changes. (d) Posttreatment facial photographs demonstrating final esthetic outcomes. (e) Posttreatment intraoral photographs showing Class I molar relationship and proper overjet. (f) Final lateral cephalometric radiograph confirming stable skeletal relationships. (g) Final panoramic radiograph showing complete eruption of permanent dentition.

Posttreatment cephalometric analysis confirmed sagittal discrepancy correction through maxillary advancement, with overcorrected molar and incisor relationships, increased overjet, and improved upper lip position. Mandibular growth remained stable during this phase (Figure 2), whereas panoramic imaging demonstrated normal dental development (Figure 2)b. Superimposition analysis revealed forward maxillary displacement (ANS and A‐point advancement) with increased maxillary length (minor palatal plane reduction anteriorly) and incisor proclination. The mandible exhibited slight posterior rotation (B‐point, Pog, and Me moving posteriorly and inferiorly) with reduced lower incisor lingual inclination. Maxillary molars moved anteriorly whereas mandibular molars showed primarily vertical movement, with minimal condylar growth (Figure 2)c.

### 3.2. Treatment Results and Long‐Term Follow‐Up

Posttreatment evaluation demonstrated significant improvement in molar, premolar, canine, and incisor relationships, with normalized overjet and increased overbite. Arch form improved substantially, with moderate dental alignment and second molar eruption initiation (Figure 2d,e). Final cephalometric records confirmed normalized maxillomandibular relationships with Class I molar and incisor positioning, proper overjet/overbite, and improved incisor inclination (Figure 2)f. Upper lip position improved relative to nasal, lower lip, and pogonion references, whereas mandibular growth followed expected pubertal patterns. All permanent teeth demonstrated normal development (Figure 2)g.

**Figure 3:**
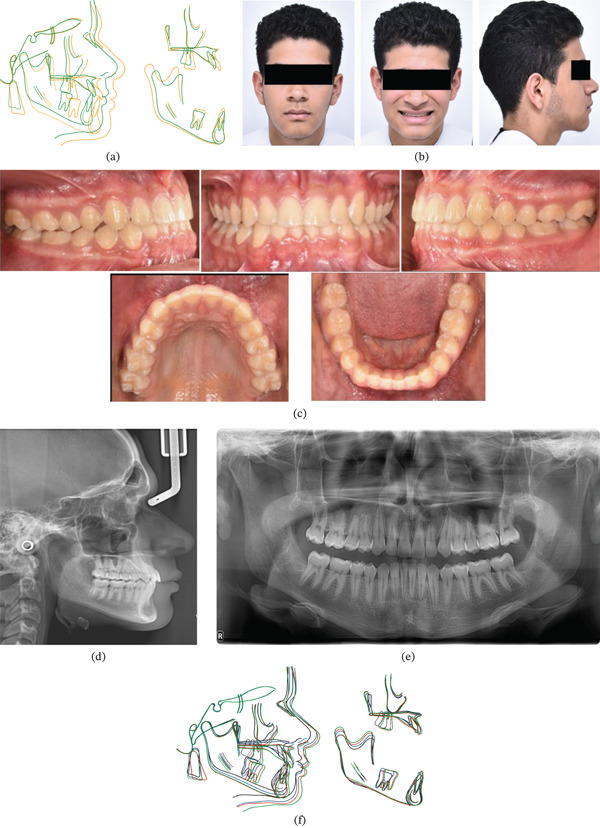
(a) Cephalometric superimposition of active treatment changes. (b) Long‐term (9‐year) follow‐up facial photographs showing maintained results. (c) Long‐term intraoral photographs demonstrating stable occlusal relationships. (d) Long‐term panoramic radiograph confirming periodontal health and root parallelism. (e) Long‐term lateral cephalometric radiograph showing postpubertal growth pattern. (f) Comprehensive cephalometric superimposition tracing growth changes through postpubertal development.

Long‐term follow‐up through the postpubertal growth phase revealed excellent stability of dental relationships with minimal changes. Anterior overjet and overbite remained stable, with well‐maintained arch forms (Figure 3a,b). Third molars were subsequently extracted, with all permanent teeth fully erupted and demonstrating excellent periodontal health (Figure 3)c. Long‐term panoramic evaluation revealed apical root resorption affecting the maxillary incisors. This finding was not evident in the immediate postorthopedic records and is most plausibly associated with the subsequent phase of comprehensive fixed orthodontic treatment rather than with early maxillary protraction therapy. This stability is interpreted considering growth‐related drift: The postpubertal reduction in ANB and overjet reflects residual mandibular growth rather than relapse, whereas stable incisor proclination contributed to occlusal maintenance.

Final cephalometric superimposition demonstrated continued maxillary elongation and anterior movement (A‐point and ANS advancing anteriorly and slightly inferiorly, with PNS moving inferiorly) accompanied by reduced angulation relative to the cranial base. Mandibular growth produced anterior‐inferior displacement of B‐point, Pog, and Me, whereas condylar growth occurred predominantly in a posterosuperior direction with gonial angle increase. Dental changes included continued upper incisor proclination with anterior movement of both upper and lower molars (Figure 3d–f).

Comprehensive evaluation of long‐term changes confirmed normalization of previously restricted maxillary growth, with both jaws following expected anteroinferior growth vectors. The mandibular plane angle increased slightly, indicating mild vertical growth tendency. Significant dental changes included sustained upper incisor proclination, whereas facial profile maintenance was achieved through proportional growth of facial thirds, resulting in labial competence and enhanced facial esthetics (Figure 3g,h).

## 4. Discussion

Early orthopedic intervention using maxillary protraction mechanics demonstrates significant benefits for Class III patients presenting with maxillary deficiency and relative mandibular excess [3–5]. Facemask therapy produces dual orthopedic effects: anterior displacement and stimulation of maxillary structures combined with reciprocal restraint and redirection of mandibular growth, resulting in favorable skeletal and dentoalveolar adaptations in both jaws [7–9]. Early interception of Class III malocclusion is particularly critical when maxillary underdevelopment represents the primary etiological factor, as untreated cases typically exhibit progressive worsening of the sagittal discrepancy due to persistent unfavorable growth patterns [9–11]. Accordingly, the present discussion emphasizes maxillary forward displacement and stimulation of nasomaxillary growth as the principal orthopedic effects of facemask therapy.

The therapeutic rationale for combining maxillary expansion with protraction involves both transverse correction and circummaxillary suture disarticulation. Although facemask therapy has demonstrated efficacy with or without rapid maxillary expansion [7–9], expansion should be reserved for cases presenting with true transverse deficiencies—particularly posterior crossbites—rather than being used solely as an adjunct for sagittal correction. In the present case, the concomitant presence of maxillary hypoplasia and bilateral posterior crossbite justified the use of rapid palatal expansion (RPE) [12–14]. The Hyrax appliance served a dual purpose, facilitating transverse expansion while providing stable anchorage for maxillary protraction. Strategic placement of labial hooks in the canine region allowed force application closer to the center of resistance of the nasomaxillary complex, consistent with Ngan et al.′s recommendations for downward‐forward force vectors of approximately 30° [15].

The treatment protocol employed a Petit‐type facemask delivering 300–500 g (approximately 16 oz) of force per side at a 30°–40° angle to the occlusal plane for 14–16 h per day. Although current evidence remains inconclusive regarding optimal force magnitude and duration, owing to considerable variability among published protocols [10], the orthopedic effects achieved during active treatment not only improved maxillomandibular relationships but also reduced compensatory dental mechanisms, such as mandibular overclosure and lower incisor retroclination. Establishing this early physiological occlusal environment facilitates more favorable dentofacial development and minimizes maladaptive dental compensation during growth [12–14].

In addition to mandibular positional changes, facemask therapy produced clinically relevant maxillary effects, including forward displacement of point A and ANS, increased maxillary length (Co–A), and improved sagittal relationship relative to the cranial base. These maxillary adaptations are central to the orthopedic correction observed in this case and support the rationale for early maxillary protraction in true skeletal Class III patients.

Functional mandibular shifts associated with anterior crossbites represent an additional critical consideration. Early orthopedic intervention effectively eliminates centric occlusion–centric relation (CO/CR) discrepancies, thereby preventing adverse functional adaptations that may exacerbate skeletal disharmony over time. In mild‐to‐moderate Class III cases, interceptive treatment may reduce or even eliminate the need for orthognathic surgery in adulthood by addressing transverse deficiencies and maximizing the remaining potential for maxillary growth [11]. The most favorable prognosis is achieved when treatment is initiated prior to the pubertal growth spurt, as untreated patients typically demonstrate progressive deterioration driven by skeletal imbalance and compensatory dental changes. Diagnostic clarification between true skeletal and functional Class III malocclusion, as well as consideration of initial severity, is therefore essential for appropriate case selection and realistic prognosis.

Posttreatment monitoring remains indispensable due to the inherent variability of craniofacial growth following maxillary–mandibular realignment. Various retention strategies, including chin cup therapy [12–14], modified Eschler appliances, Class III functional appliances, and fixed orthodontic mechanics with Class III elastics, have been advocated to modulate residual mandibular growth and minimize relapse [13–15]. Gallagher et al. emphasize the importance of intentional overcorrection toward a Class II relationship, noting that the establishment of positive overjet and overbite constitutes a key factor for long‐term stability. Baccetti et al. demonstrated that treatment initiated during the early mixed dentition produces more favorable craniofacial adaptations than late intervention, although different relapse tendencies may affect maxillary sagittal position in early‐treated cases and mandibular position in late‐treated patients. Particular attention should also be given to mandibular rotation patterns during treatment, as downward‐backward rotation may predispose to subsequent horizontal growth expression and potential relapse [14].

Apical root resorption observed during long‐term follow‐up represents a recognized potential adverse effect of comprehensive fixed orthodontic mechanics. Its identification in this case underscores the importance of differentiating orthopedic effects from those related to subsequent orthodontic treatment and highlights the need for careful radiographic monitoring during prolonged treatment protocols.

Significant soft tissue improvements accompanied the skeletal changes observed in this case, including profile straightening, improved lip competence [15], and enhanced facial balance. Optimal timing for orthopedic intervention often coincides with eruption of the maxillary central incisors, facilitating early anterior occlusal stabilization. During the 9‐year follow‐up, postpubertal growth changes were carefully evaluated to distinguish between relapse and growth‐related drift. Although the ANB angle decreased to 0.9° and overjet was reduced from 4.2 to 2.5 mm, these changes are consistent with expected residual mandibular growth rather than loss of orthopedic correction, supporting the long‐term stability of the treatment outcome. For younger patients, chronological age and dental development guide intervention timing, whereas cervical vertebral or hand‐wrist maturation indicators prove more appropriate for older children [7].

## 5. Conclusion

This 9‐year follow‐up case demonstrates the effectiveness of combined Hyrax expansion and facemask protraction in managing Class III malocclusion with maxillary hypoplasia and mandibular prognathism. Successful growth modification yields functional, esthetic, and psychosocial benefits, though long‐term monitoring, particularly through growth peaks using modified Type III functional appliances, remains essential to prevent relapse. As illustrated in this case, patient compliance and parental cooperation constitute critical success factors for achieving stable, favorable outcomes.

## Author Contributions

Conceptualization, Jenny Angélica Saldarriaga‐Valencia, Ary dos Santos‐Pinto, and Carlos M. Ardila; methodology, Jenny Angélica Saldarriaga‐Valencia, Ary dos Santos‐Pinto, and Carlos M. Ardila; investigation, Jenny Angélica Saldarriaga‐Valencia, Ary dos Santos‐Pinto, and Carlos M. Ardila; data curation, Jenny Angélica Saldarriaga‐Valencia, Ary dos Santos‐Pinto, and Carlos M. Ardila; writing—original draft preparation, Jenny Angélica Saldarriaga‐Valencia, Ary dos Santos‐Pinto, and Carlos M. Ardila; writing—review and editing, Jenny Angélica Saldarriaga‐Valencia, Ary dos Santos‐Pinto, and Carlos M. Ardila.

## Funding

No funding was received for this manuscript.

## Disclosure

All authors approved the final version and are accountable for the work.

## Consent

Following detailed discussion of treatment alternatives, risks, and benefits with the patient and parents, written informed consent was obtained. This consent specifically included permission for the publication of the patient′s identifiable images within this manuscript.

## Conflicts of Interest

The authors declare no conflicts of interest.

## Data Availability

The datasets used and/or analyzed during the current study are available from the corresponding author.
